# Implementing the Debriefing Assessment for Simulation in Healthcare (DASH) Tool for Training Medical Faculty

**DOI:** 10.7759/cureus.69290

**Published:** 2024-09-12

**Authors:** Thura Al-Khayat, Stefanie Carter, McHenry Mauger, Aman Patel, Krina Patel, Lilia Chavarria

**Affiliations:** 1 Medical Education, Nova Southeastern University Dr. Kiran C. Patel College of Allopathic Medicine, Fort Lauderdale, USA; 2 Faculty Affairs, Nova Southeastern University Dr. Kiran C. Patel College of Allopathic Medicine, Fort Lauderdale, USA; 3 Biostatistics, Nova Southeastern University Dr. Kiran C. Patel College of Allopathic Medicine, Fort Lauderdale, USA; 4 Medicine, Nova Southeastern University Dr. Kiran C. Patel College of Allopathic Medicine, Fort Lauderdale, USA; 5 Simulation, Broward College Health Sciences Simulation Center, Davie, USA

**Keywords:** debrief, experiential learning, faculty development, healthcare simulation, patient safety, prebrief, simulation-based-education, • simulation in medical education

## Abstract

Introduction

Despite simulation being widely recognized as an essential component of medical education, there remains a dearth of literature on its implementation and effectiveness in pre-clerkship medical education. In order to effectively encompass healthcare simulations within medical instruction, it is imperative to first address the often neglected gap in faculty development and training. With this in mind, we aim to present our experience in implementing the Debriefing Assessment for Simulation in Healthcare (DASH) tool in training faculty at Nova Southeastern University Dr. Kiran C. Patel College of Allopathic Medicine, Fort Lauderdale, USA.

Methods

Twenty participating faculty practiced instructing a simulation session and completed the DASH tool. Quantitative and qualitative approaches were used to report the findings.

Results

The Survey Rater and Student DASH version results were analyzed using the Mann-Whitney U test to see if there was a difference between the two groups for each question.

Conclusions

All faculty participating in healthcare simulations should be trained in order to deliver a structured debriefing experience. In healthcare medical simulation, the identification of faculty training is imperative to address many of the challenges of healthcare today, including patient safety issues and limitations of medical training in hospital settings.

Implications

The study aims to serve as a template that researchers can use in identifying potential faculty training development, as the benefits of having faculty trained in simulation methodology are successful in higher educational settings. Further studies in simulation-based education faculty training are recommended.

## Introduction

The use of simulation as an activity has been reported for centuries. During the 18th century, simulation use for healthcare training evolved and continued to grow throughout the years. It took a decline in the early 20th century and then picked up again during the 1960s. During that time, Åsmund S. Lærdal created Resusci Anne to teach proper basic life support techniques. It eventually became famous for paving the way for using simulators for the specific purpose of healthcare education. Training healthcare professionals on patient safety using simulation was later introduced in 1990 by David Gaba and his team. In addition to the ultimate goal of patient safety, the use of high-technology simulators addressed the issues of patient sensitivity when used as subjects for training [1]. Technological advances continued to grow steadily, and manufacturers continued to innovate and advance in creating diverse modern medical simulation technologies that serve as training aids in various areas of simulation in healthcare [2].

Simulation-based education (SBE) continues to increase and spread as an effective method of teaching and training healthcare professionals among various institutes and has been integrated into the healthcare curriculum [3]. In 2011, the Association of American Medical Colleges, the Society for Simulation in Healthcare, the Association for Standardized Patient Educators, and the American Association of Colleges of Nursing released the results of a 2010 survey. The survey revealed that medical schools in North America adopt SBE for learner assessments more prevalently than teaching hospitals, with adoption rates of 68% versus 25%, respectively [4]. This year the Simulation in Healthcare journal published an article describing the current trends of SBE in undergraduate medical education since the 2011 survey. They confirmed the ongoing high prevalence of more than 90% SBE adoption in allopathic and osteopathic medical schools, whether procedural or not [5]. Despite simulation education being widely recognized as an essential component of medical clerkship, residency, and nursing programs, there remains a dearth of literature on its implementation and effectiveness in medical pre-clerkship. In order to effectively encompass healthcare simulations within medical instruction, it is imperative to first address the often-neglected gap in faculty development and training. SBE offers a dynamic approach to training health professions students. These important experiences allow students to engage in realistic clinical scenarios before entering the clinical environment. The effectiveness of the programming is contingent on how prepared and experienced the faculty are that facilitate the simulation cases. Therefore, the importance of faculty development programming tailored to prepare educators to deliver high-quality simulation training is essential. This paper aims to present our experience in implementing the Debriefing Assessment for Simulation in Healthcare (DASH) tool in training faculty at the Nova Southeastern University Dr. Kiran C. Patel College of Allopathic Medicine (NSU MD).

The DASH in simulation refers to the “Debriefing Assessment for Simulation in Healthcare,” which is a tool used to evaluate the quality of debriefing sessions in healthcare simulation training. The tool was developed and validated by the Center for Medical Simulation (CMS) and consists of six elements that assess the various components of debriefing, such as establishing a safe learning environment, promoting reflection and discussion, and providing feedback on performance [6]. The primary objective of the DASH intervention is to enhance the faculty’s preparation to teach in a simulated environment, which would in turn enhance the implementation and evaluation processes of simulation in medical education.

Simulation professional development is deemed necessary for the success of simulation-based learning and is often limited [7]. As SBE advances, a sustained emphasis on faculty development will be pivotal to ensure its continued success in medical education [8]. According to the Healthcare Simulation Standards of Best Practice guidelines, a simulation must have defined goals and objectives, a sufficient prebrief, an established case scenario, and adequate debriefing. In this study, we covered all parts of the simulation event as described in the Healthcare Simulation Standards of Best Practice guidelines.

This research aimed to identify areas for improvement in faculty development for simulation training, utilizing the DASH tool developed by the CMS. The key research question was: What is the relationship between the student version and the rater version of the DASH survey responses when assessing faculty performance in simulation-based education?

This project was accepted for poster presentation at two conferences: the International Conference on the Future of Health Professions Education on November 3, 2022, and the International Meeting for Simulation in Healthcare on January 22, 2023.

## Materials and methods

Sample population and survey administration

The sample population was obtained from volunteers meeting certain criteria for study participation. The study aimed to implement the DASH tool in training faculty at NSU MD in facilitating simulation sessions; therefore, the potential participants had to be located within the NSU MD pool and must hold a terminal degree to be able to serve as facilitators. Upon obtaining approval from the Nova Southeastern University Institutional Review Board (IRB number 2022-4 Institutional Review Board Dr. Kiran C. Patel College of Allopathic Medicine), volunteers were recruited via e-mail. The invitation to participate in the simulation training was facilitated by the Office of Faculty Affairs in January 2022, with a total reach of approximately 70 full-time faculty. The researcher also contacted potential participants via staff meetings. Over the period from January 2022 to August 2022, over seventy potential volunteers were contacted, and 22 responded. One faculty member participated in the workshop and chose not to participate in the study. Another member observed and completed the pre-training survey but did not actively participate in the workshop due to time restraints. This left a total of 20 participants in the workshop and study. Out of the 20 participants who completed the pre-training survey, 16 (80%) read the pre-workshop prep material, and 14 (20%) did not. Eighteen participants completed the post-training survey.

Consent forms were distributed to the participants, whether electronically or physically, and all forms were scanned and saved with the PI in a confidential folder. Participants were provided with the required prereading material on healthcare simulation that described the elements of DASH using the DASH Rater Handbook [9] and the Promoting Excellence and Reflective Learning in Simulation (PEARLS) tool for debriefing [10]. The PEARLS framework, which stands for Promoting Excellence And Reflective Learning in Simulation, is a reliable tool that encourages an engaging and safe debriefing learning environment. Participants were also asked to review a prerecorded demonstration of a simulation session that was prepared and provided by staff and faculty at the Broward College Simulation Center. The time from initial participant contact to completion of data collection was six months. Anonymous pre- and post-surveys were submitted to each volunteer via Microsoft forms; once completed, the surveys were sent online directly to the researcher’s portal, which can only be accessed by the researcher. The DASH scores were obtained during the training at the simulation center located at Broward College, as discussed under study design. Participants were granted a Certificate of Completion by the Health Sciences Simulation Center at Broward College.

Survey development

Identified domains in the pre-workshop survey were relevant demographics that included graduate degrees and current rank, in addition to the courses they were involved in teaching at NSU MD (Table 1). Both the pre- and post-surveys consisted of questions used for comparison of the experience; these included faculty’s familiarity with simulations in healthcare and familiarity with the PEARLS debriefing method and the DASH assessment tool, in addition to their comfort level of creating a simulation session. The post-workshop survey also included open-ended qualitative questions about the training session: how helpful it was, its strengths, and how it can be improved. Likert scale and open-text questions were used. To address content validity, the survey questions were developed by the PI and reviewed by the second author, who is a content expert in healthcare simulations. Both came to an agreement on the relevance of including each question.

**Table 1:** Graduate degree and rank of participating faculty

Graduate degree	Number of faculty	Percentage
MD/MD-PhD/DO	8	40
PhD	12	60
Rank		
Assistant Professor	7	35
Associate Professor	7	35
Professor	4	20
Adjunct	2	10

Study design and participants

Participating faculty practiced instructing a simulation session and were assessed using the DASH tool.

Two medical scenarios were used for the simulation training, both developed by the faculty at Broward College Simulation Center and modified by the PI and MD who serve as faculty at NSU MD. One scenario was delivered via a high-fidelity manikin, Laerdal Sim Man 3G, and the other was delivered via a standardized patient.

Participants worked in groups, taking turns either being the “instructor” or the “student.” The instructor was provided with the case before the session began, was asked to review it, and was provided with clarifications on any inquiries they had. The session began with a brief conducted by the instructor, followed by the simulation that was conducted by the “students” and facilitated by the instructor in the control room, and ended with the instructor debriefing the students using the PEARLS debriefing tool. The entire framework was observed by the “rater (debriefer),” who also debriefed the instructor and gave verbal feedback on their performance. Participants were asked to complete the DASH scoresheet forms that asked trainees to rate behaviors associated with each of the six DASH elements.

For the purpose of this study, the researchers chose to identify the relationship between the Rater and Student versions of the DASH scores [11-13]. Within each element, the DASH survey debriefing may range from outstanding to detrimental, with a rating of 1 (Extremely Ineffective) to 7 (Extremely Effective /Outstanding) on a Likert scale.

A 100% DASH completion rate was achieved for the 20 who participated in the training. No seen or unforeseeable effects were evident at any stage of the research on either participants or data during the collection or analysis portions of the study.

The DASH surveys were scored by simulation staff at the Broward College Simulation Center who either held certifications as Certified Healthcare Simulation Educators or were faculty members working with simulation for at least a two-year period. A Microsoft Excel spreadsheet was created within which data was entered for each of the 20 participants for the total scores of each DASH survey (Rater Version and Student Version). Paper files and data were securely stored in a locked drawer in the office of the PI. Digital files and data were securely filed in the computer of the PI. All data, whether digital or hard copies, were deidentified.

We employed a robust statistical approach, utilizing the Mann-Whitney U test to see if there was a difference between the two versions (Rater and Student) for each question. A boxplot for each question was created to allow a visual distribution of how the questions were answered, with the use of important statistical measures such as the median, quartiles, and potential outliers, in addition to allowing visual distributions between the two groups: raters and students. JMP Pro 16/R 4.2.2 was used for all data analysis.

## Results

Survey results

Table 1 shows the graduate degrees of NSU MD faculty participating in the workshop held in addition to their ranks. The courses participating faculty were involved in teaching are Practice of Medicine (POM) 1,2,3, Fundamentals, Heme, Gastrointestinal, Nutrition, Endocrine, Reproductive (GINER), Cardiovascular, Pulmonary, Renal (CPR), Brain, Body, Behavior (BBB), Practice-Based Learning/ Inquiry cases (PBL/IQ), Master of Biomedical Sciences (MBS), Dental, Clerkships (Surgical/OBGYN/Peds), Clinical Skills and Reasoning - Diagnostic Medicine (CSR-DM), Transition to Residency (TTR), Anatomy, and Health Care Sciences.

Table 2 describes how familiar faculty members were with healthcare simulations before the workshop.

**Table 2:** Level of familiarity faculty members had with healthcare simulations prior to the workshop

Familiarity with simulations in healthcare before the workshop	Number of faculty	Percentage
Very unfamiliar	3	15
Somewhat unfamiliar	3	15
Neither familiar nor unfamiliar	0	0
Somewhat familiar	9	45
Very familiar	5	25

**Figure 1:**
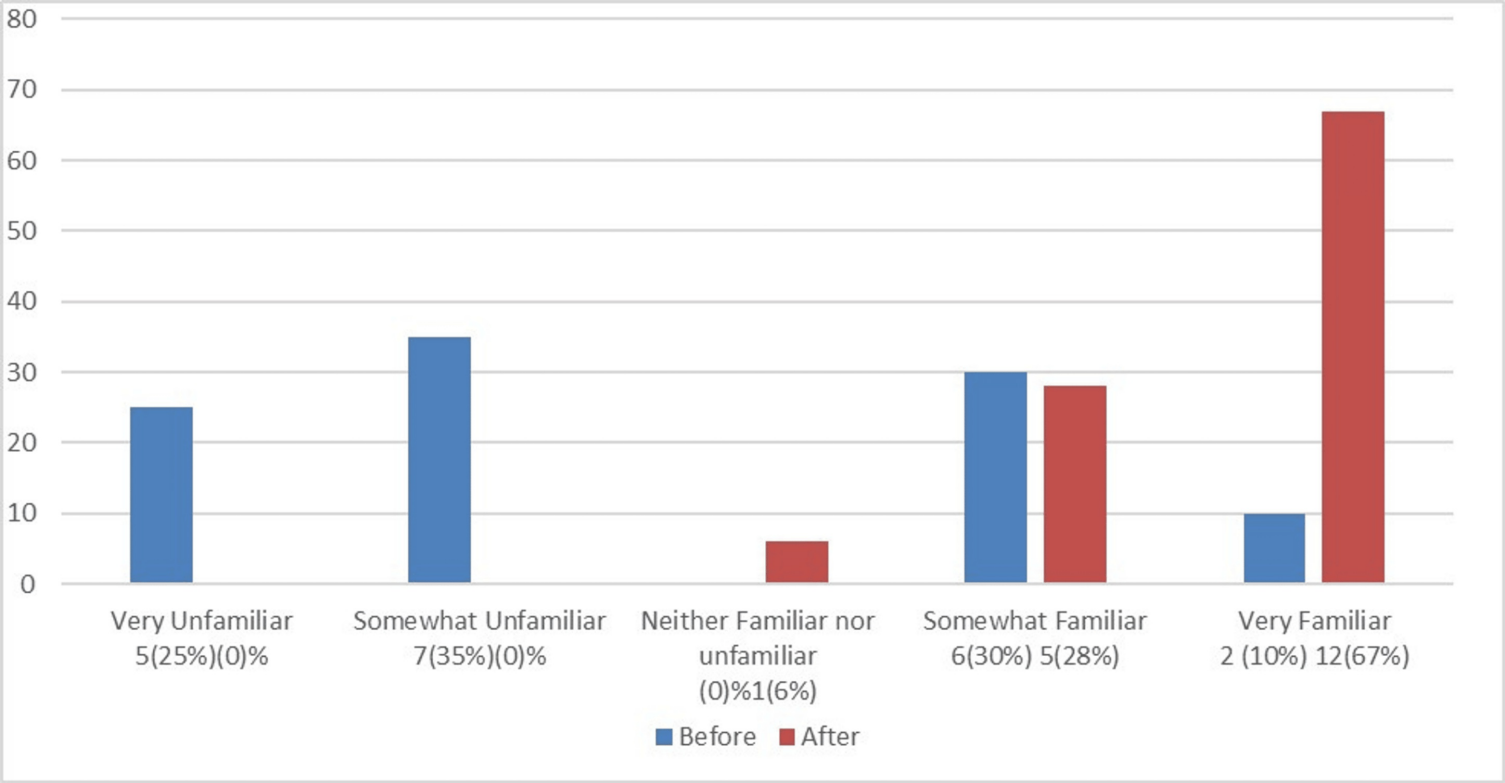
Comparison of NSU MD faculty’s familiarity with the DASH model before and after the workshop The total number of participants was 20 before the workshop and 18 after. DASH, Debriefing Assessment for Simulation in Healthcare; NSU MD, Nova Southeastern University Dr. Kiran C. Patel College of Allopathic Medicine

**Figure 2:**
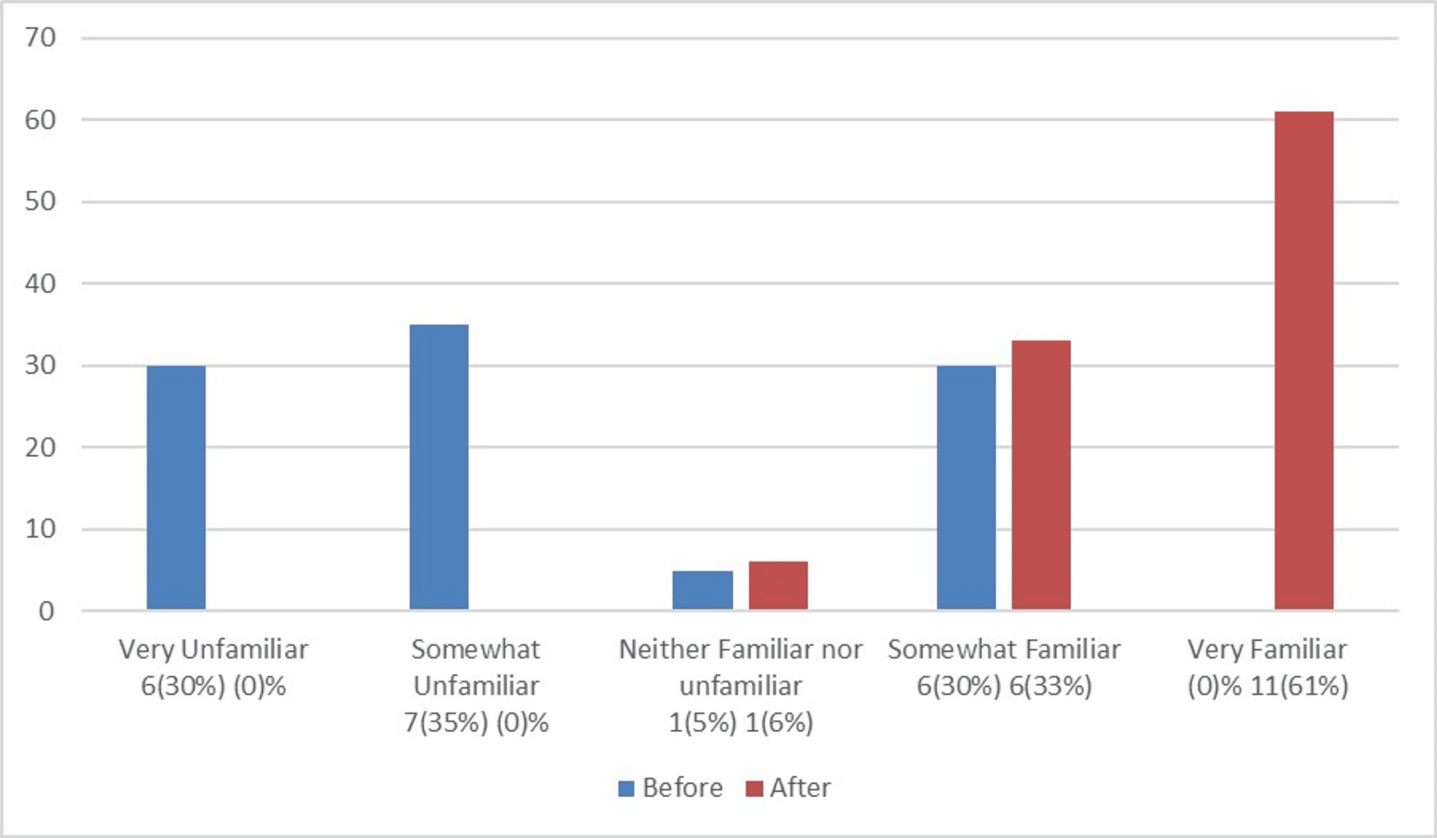
Comparison of NSU MD faculty’s familiarity with the debriefing tool PEARLS before and after the workshop The total number of participants was 20 before the workshop and 18 after. NSU MD, Nova Southeastern University Dr. Kiran C. Patel College of Allopathic Medicine; PEARLS, Promoting Excellence and Reflective Learning in Simulation

**Figure 3:**
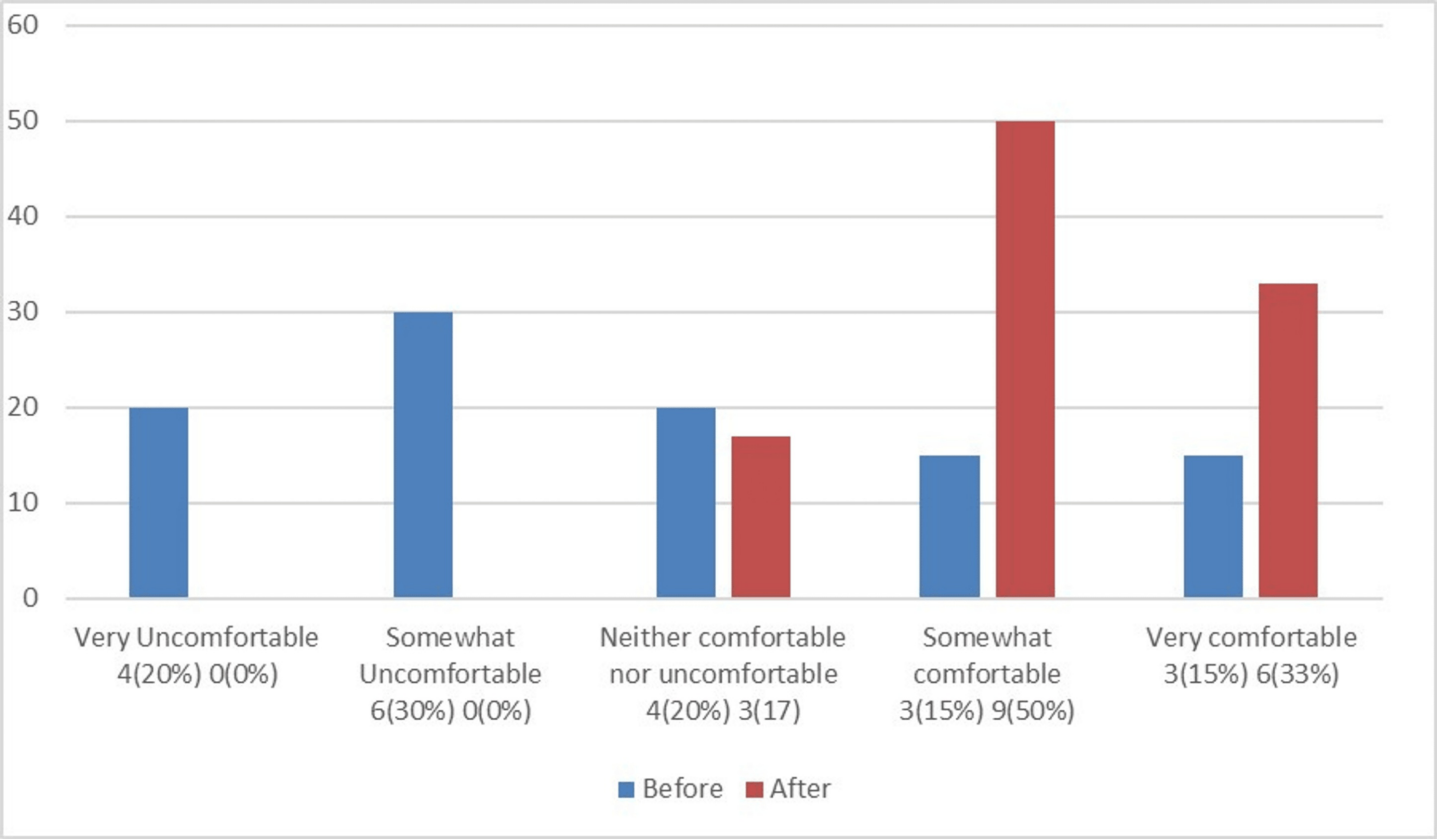
Levels of comfort among NSU MD faculty with creating a simulation session before and after the workshop The total number of participants was 20 before the workshop and 18 after. NSU MD, Nova Southeastern University Dr. Kiran C. Patel College of Allopathic Medicine; PEARLS, Promoting Excellence and Reflective Learning in Simulation

**Figure 4:**
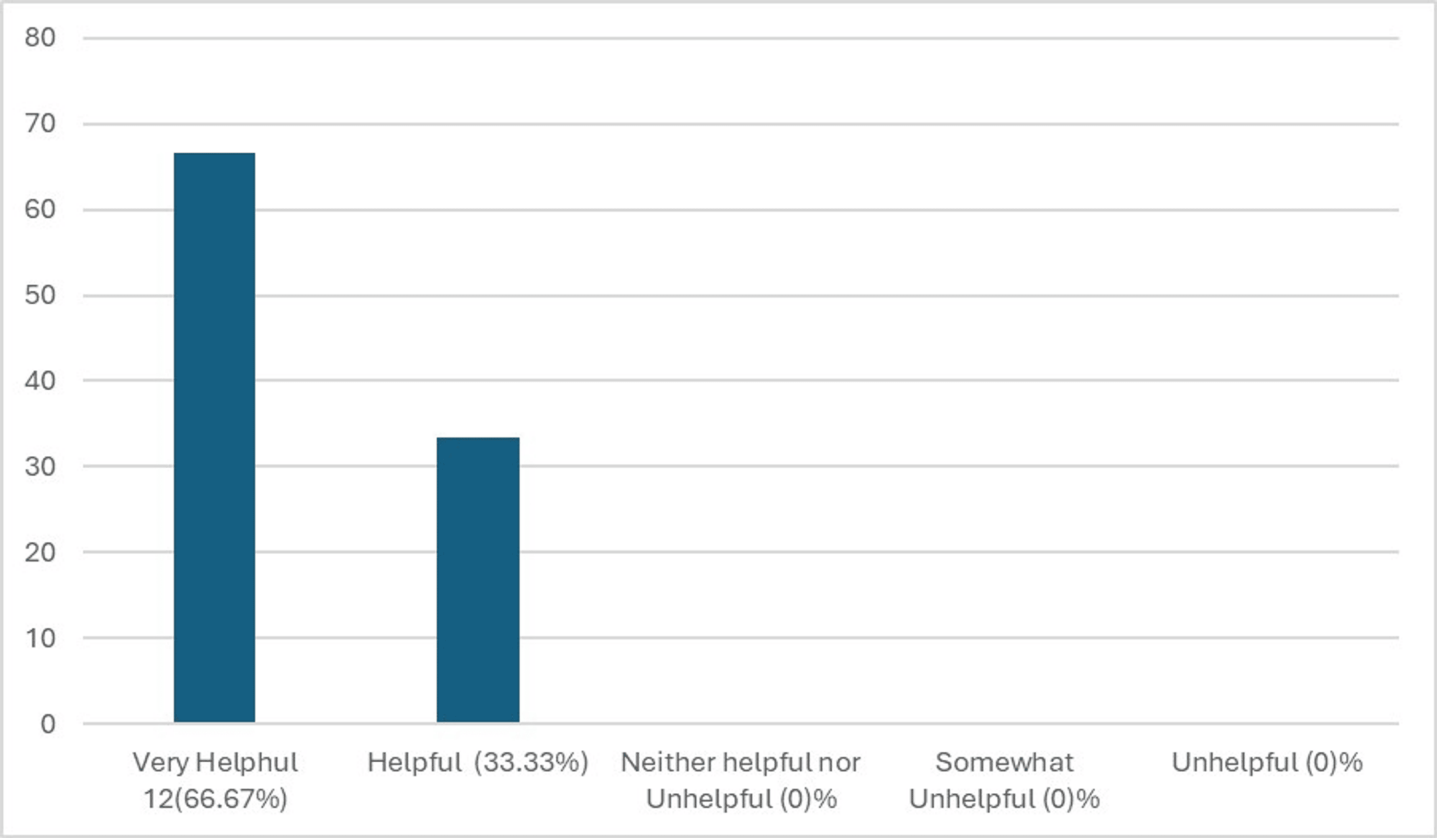
Participant feedback on the helpfulness of the workshop Out of 18 NSU MD faculty who completed the post-training survey, 12 (66.67%) found the workshop very helpful, and six (33.33%) found it somewhat helpful. No participants rated the workshop as very unhelpful, somewhat unhelpful, or neither helpful nor unhelpful. NSU MD, Nova Southeastern University Dr. Kiran C. Patel College of Allopathic Medicine

Figure 1 compares the familiarity of NSU MD faculty with the DASH Model before and after the workshop, while Figure 2 assesses their familiarity with the debriefing tool PEARLS before and after the workshop. Figure 3 illustrates how comfortable NSU MD faculty were with creating a simulation session before and after the workshop. Figure 4 shows that, out of the 18 participants who completed the post-training survey, 12 (66.67%) found the workshop very helpful, six (33.33%) found it somewhat helpful, and none rated it as very unhelpful, somewhat unhelpful, or neither helpful nor unhelpful.

Quantitative statistical analysis

Table 3 describes the summary statistics that show the results of the survey between Rater and Student. The P-values are a result of a Mann-Whitney U test to see if there was a difference between the two groups for each question. The authors find that the two groups only had a significant difference (P < 0.05) in question 1 (P = 0.024). Thus, the results demonstrated that the prebriefing portion of the simulation could be enhanced. Rater vs. Student had significant differences in perception of the prebriefing stage of the simulation activity. The participants who played the students during the study perceived that the prebriefing encompassed most elements of the survey. However, the rater did not, thus prebriefing could be enhanced. DASH element 1 is the prebrief script that was used.

**Table 3:** Summary statistics showing the results of the DASH survey comparing the Rater and Student versions The Mann-Whitney U test was used to determine the differences between the two versions of each question. The rating scale is as follows: 1 = Extremely Ineffective / Detrimental, 2 = Consistently Ineffective / Very Poor, 3 = Mostly Ineffective / Poor, 4 = Somewhat Effective / Average, 5 = Mostly Effective / Good, 6 = Consistently Effective / Very Good, 7 = Extremely Effective / Outstanding. DASH Rater Version questions (elements): Element 1: establishes an engaging learning environment; Element 2: maintains an engaging learning environment; Element 3: structure the debriefing in an organized way; Element 4: provokes engaging discussion; Element 5: identifies and explores performance gaps; Element 6: helps trainees achieve or sustain good future performance. DASH Student Version questions (elements): Element 1: The instructor set the stage for an engaging learning experience; Element 2: The instructor maintained an engaging context for learning; Element 3: The instructor structured the debriefing in an organized way; Element 4: The instructor provoked in-depth discussions that let me reflect on my performance; Element 5: The instructor identified what I did well or poorly and why; Element 6: The instructor helped me see how to improve or how to sustain good performance.

	Rater (N = 20)	Student (N = 20)	P-value
Question 1			0.024*
Mean (SD)	5.70 (0.733)	6.25 (0.716)	
Median [Min, Max]	6.00 [5.00, 7.00]	6.00 [5.00, 7.00]	
Question 2			0.067
Mean (SD)	5.65 (0.875)	6.15 (0.988)	
Median [Min, Max]	5.50 [4.00, 7.00]	6.00 [4.00, 7.00]	
Question 3			0.43
Mean (SD)	5.90 (0.852)	6.10 (0.912)	
Median [Min, Max]	6.00 [4.00, 7.00]	6.00 [4.00, 7.00]	
Question 4			1
Mean (SD)	5.70 (1.08)	5.70 (1.08)	
Median [Min, Max]	6.00 [4.00, 7.00]	6.00 [4.00, 7.00]	
Question 5			0.943
Mean (SD)	5.80 (1.24)	5.85 (0.988)	
Median [Min, Max]	6.00 [3.00, 7.00]	6.00 [4.00, 7.00]	
Question 6			0.619
Mean (SD)	5.80 (1.15)	6.00 (1.03)	
Median [Min, Max]	6.00 [3.00, 7.00]	6.00 [4.00, 7.00]	

In question 2, we see a borderline significance (P = 0.067) between the two groups, so we consider those differences.

**Figure 5:**
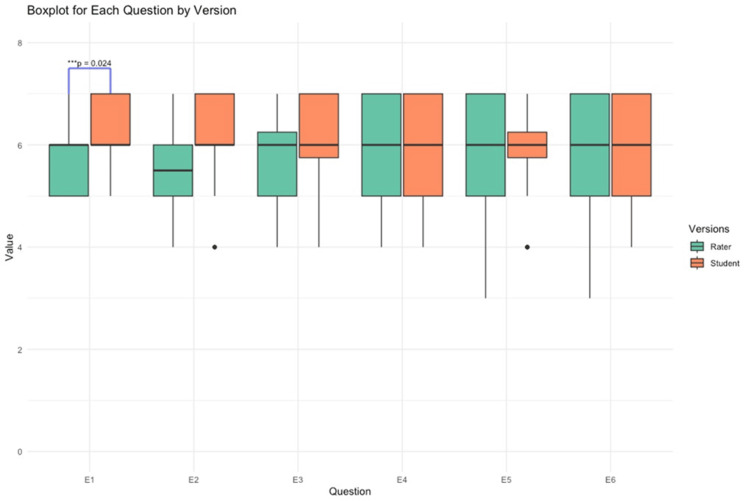
Boxplot displaying the distribution of responses for each question, including the median, quartiles, and potential outliers The plot also compares the distributions between the two groups: raters and students. Data analysis was performed using the Mann-Whitney U test with JMP Pro 16/R 4.2.2 software.

A boxplot (Figure 5) is provided to display the results of the summary statistics table in graphic form. This form graphic gives us the best way to visually see the distribution of how they answered each question, with the use of important statistical measures such as the median, quartiles, and potential outliers. Additionally, it gives us a way to see the distributions between two groups: raters and students. JMP Pro 16/R 4.2.2 was used for all data analysis.

Question 1

Rater: The median is 6, with an IQR indicating moderate variability. In this boxplot, the range is from 5 to 7.

Student: The median is also 6, and their range is the same, but we can see in the boxplot that their scores were higher.

Comparison: We found that there was a statistically significant difference between the students and the rater in this question (P = 0.024), with students generally having a higher score. The boxplot visually supports this with a higher mean and slightly less spread for students.

Question 2

Rater: The median is 5.50, and we can see a wider range in the whiskers that go from 4 to 7. There is a noticeable lower outlier at 4.

Student: The median is 6, with a higher mean and median, so we see there is slightly more variability.

Comparison: The P-value of 0.067 shows that the statistically significant difference was borderline, and it can be seen visually in the boxplot that it is like Question 1.

Question 3

Rater: The median is 6, with moderate variability. The whiskers extend to 4, indicating a broader range.

Student: The median is also 6, with a slightly higher mean and a longer whisker, indicating slightly more variability.

Comparison: Both groups have matching medians, but students show slightly more variability and a higher mean, which means it was more consistent and had slightly higher ratings.

Question 4

Rater: The median is 6, with a range that goes from 4 to 7, which indicates a broad spread.

Student: The median here is also 6, and we see there is matching scoring as seen in the boxplot and the summary statistics.

Comparison: Both groups show matching statistics and boxplot distributions, showing consistent responses between both groups.

Question 5

Rater: The median is 6, with a wider IQR and longer whiskers going to 3, meaning slightly more variability.

Student: The median here is also 6, with shorter whiskers and fewer lower values compared to raters.

Comparison: Students show slightly less variability and higher scoring with fewer outliers; the boxplots showed a wider distribution for raters.

Question 6

Rater: The median is 6, with moderate variability indicated by the IQR and whiskers extending to 3, including an outlier at the lower end.

Student: The median here is also 6, and there is less variability with fewer lower scores.

Comparison: Both groups have similar medians, but students show slightly higher ratings with scores not being low, which can be seen with the shorter whiskers and higher mean.

Symmetry and skewness: Looking at the medians from a macro, the students and raters were generally scoring 5-6 and were centered within the boxes for both groups, meaning we may have symmetric distributions. We see some skewness in some of the questions, but the responses felt similar.

Spread and variability: In looking at the boxplots, we can see that the students tend to show less variability in their responses compared to Rater. This can be confirmed in our plots, with distance in the quartiles and the medians staying the same for all the questions.

Qualitative component

Qualitative analysis was done manually without the use of a database using the post-survey narrative questions. The investigators read through the answers to each narrative question to identify common themes. These themes were then color-coded to clearly visualize those that were most repetitive and were more focused by the participants.

## Discussion

Quantitative analysis

The results demonstrated that during the prebriefing session of a simulation, faculty members should include more information and better prepare students for the simulation event. While participants perceived that the prebriefing encompassed most elements required in the survey, the rater noted that additional information should have been included during the prebriefing session. In this study, prebriefing is an area that could be improved based on the rater’s perspective. Prebriefing serves to prepare and orient learners to the simulation experience. Providing learners with an adequate orientation and creating a psychologically safe environment lowers the participant’s fear and anxiety [14].

Prebriefing is known as the orientation of the simulation scenario. During the prebriefing phase, faculty should introduce themselves, describe the simulation environment, and define expectations for the simulation activity, including learning objectives. The faculty should also clarify the strengths and weaknesses of the simulation and how the students could get the most out of simulated clinical experiences. The faculty should also describe the logistical details. Finally, during the prebrief, faculty should stimulate students to share their thoughts and questions and feel that they would not be shamed or humiliated in the process.

Prior research has demonstrated that prebriefing is an important part of the simulation scenario for optimal simulation performance and effective debriefing [15]. The overall simulation literature has placed significant emphasis on prebriefing, which involves sharing information with participants to enhance psychological safety and engagement. This process is particularly important for the session that typically follows immediately after the prebriefing. Key actions recommended for effective prebriefing include clarifying objectives, ensuring equipment functionality, defining the roles of participants and faculty, maintaining session confidentiality, and setting clear expectations for participants during the simulation [16]. It is during the prebrief phase that students can develop situation awareness and develop mental models that become the foundation for understanding perceived information that will be applied to actions during the scenario. The connection of prior knowledge and experience begins with the prebrief [17]. It is during prebriefing that a fictional contract is established to suspend disbelief and recognize the simulation environment’s limitations. During the prebriefing, a shared mental model agreement is created that both the learner and faculty member have obligations to each other: the faculty in observing the student and the student engaging in the case’s clinical problem [18]. Another previous study demonstrated that prebriefing for high-fidelity simulation in nursing education resulted in a larger effect on collaboration among participants (0.82 vs. 0.27, P = 0.004) [19]. According to the Healthcare Simulation Standards of Best Practice, an effective and well-designed prebriefing will create a psychologically safe learning environment, prepare and engage simulation learners, and pave the way for a more effective debriefing [20]. Chamberlain (2017) study supports the utilization of prebrief as an effective tool in simulation learning. Chamberlain's study revealed that nursing students who received orientation prior to simulation activities were more apt to listen to given cues and apply tasks accordingly during the simulation scenario. In addition, in order to improve the student’s learning and engagement, prebriefing should include enough time to identify the roles and responsibilities of each participant [21].

Thus, the findings underscore the importance of enhancing the prebriefing session in simulations. While participants generally felt that the prebriefing covered the necessary elements, the rater identified areas where additional information could significantly benefit the preparation process. This study highlights the critical role of prebriefing in SBE, emphasizing the need for faculty to provide comprehensive and detailed information to better prepare students. Effective prebriefing fosters a psychologically safe environment, clarifies objectives, defines roles, and ensures equipment functionality, all of which are essential for optimal simulation performance and effective debriefing.

Enhancing prebriefing practices is crucial for preparing and orienting learners, ultimately optimizing the overall simulation experience and improving educational outcomes. Continuous faculty development is recommended for instructors to add the necessary skills to deliver a structured prebriefing experience.

Qualitative comments

Narrative Question 1: What Are the Strengths of the Training Session?

Participants had a mixed background, some of whom had prior experience in SBE and others who did not. For both groups, the responses were the same; the most common theme that arose under this question was the value of putting oneself in a student’s position, which provided an insight into how students may feel during SBE.

It allows faculty to become familiar with simulation and to have the experience of being the facilitator and the student. I think it is important the faculty play the role of the student because it allows the faculty to be in the student's shoes and see firsthand the challenges the students have, and as a result, faculty can incorporate the experience they have playing the student into their role as a facilitator

Learning by doing

This was a very helpful exercise to put ourselves in the student’s shoes during the simulation as well as observe our colleagues in a similar role. This provided me with insight into the emotional and mental processes that my students may experience when they are placed in a simulation environment

There is very limited research, close to nonexistent in medical education that looks into this methodology of training by having faculty play the role of the learner. This proved to be an effective method, as seen from the comments above, from which the faculty gained insight into the learners’ perspectives and how students received input and feedback. This enabled an environment of understanding and encouraged faculty to deliver feedback with empathy and kindness. The power of kindness and using a nonjudgmental tone when giving feedback has been looked into, as reported in a study by Kumar and Epley, in which both givers and recipients of acts of kindness reported positive feelings as a result of these acts, no matter how trivial they may seem [22].

Narrative Question 2: What Could Be Done To Improve the Training Session?

When developing the faculty training model, the investigators considered numerous factors. A significant aspect was the time constraints and faculty commitments to teaching, service, and other assigned duties, which presented a limitation of the study. To address this, the pre-reading material was condensed into PowerPoint slides and emailed to participants for review before their scheduled workshop dates. Additionally, the staff and faculty created a video recording of an SBE session, including the debriefing, which was provided to participants in advance.

However, studies, including those by Jeffries et al., have recommended dedicating specific time for faculty development sessions in simulations, ideally spanning three to four days. This approach would offer participants ample opportunity to practice and learn, thereby enhancing their ability to integrate simulations into their curriculum effectively [23].

Narrative Question 3: Any Additional Feedback You Would Like To Add?

Faculty participants agreed that they would like to have further training sessions in SBE not only to further enhance their skills but also to maintain the skill sets they learned from the workshop.

Would love to have an ‘advanced skills session’ for faculty with some degree of simulation experience to further enhance their skills (continuing faculty development)

Ongoing observation and training of instructors would be appreciated to maintain the skill set and goals of the program

These comments did not come as a surprise, as medical school teaching faculty continuously strive to improve through faculty developments to provide the best learning experience for their students. Pannekoeke et al. conducted a continuing professional development for SBE facilitation in a small school and concluded that even with limited resources, this contributed to developing and maintaining facilitator competence and confidence in SBE, highlighting the importance of ongoing training [7].

A structured, safe learning environment along with learning from the experiences of others becomes the foundation of SBE collaboration and self-reflection. SBE provides a medium for immediate and actionable feedback for the learner to grow from, thus enhancing/bolstering the process of self-reflection.

In summary, further studies in SBE faculty training are recommended. Future faculty development on simulations in healthcare at NSU MD is suggested using the criteria listed in Healthcare Simulation Standards of Best Practice^TM^ Simulation Design [24].

Limitations

Two major limitations of this study could be addressed in future research. These were the limited number of participants and the short time of training that the workshop provided. Both limitations can be attributed to the time constraints NSU MD faculty have and to their various additional commitments and responsibilities.

To overcome this, staff and faculty at Broward College Simulation Center recorded a demonstration of a simulation that was distributed to the participants before the session. This was in conjunction with the document that they were provided with beforehand on Simulations in Healthcare and references to the DASH and PEARLS tools.

## Conclusions

The main purpose of this study was to implement the DASH tool in training for the Nova Southeastern University MD program. In healthcare medical simulation, the improvement of faculty training is imperative in addressing many of the challenges to healthcare education, including patient safety issues and limitations of medical training in hospital settings. The process for producing an effective faculty development program to support simulation training includes conducting a needs assessment, strategic program design, and resource allocation, in addition to continuously evaluating the effectiveness of the faculty development through participant feedback, performance assessments, and learner outcomes. Feedback is essential to refine the program and address any possible changes. Further, there is an opportunity to provide ongoing support to faculty through professional development and mentorship. Identifying additional resources globally and creating a network of simulation educators that can contribute to knowledge sharing and cross-institutional collaboration can also improve the training of faculty to facilitate simulation in medical education.

Our experience in preparing faculty for SBE provides valuable insights applicable to institutions aiming to educate their faculty in simulation. Emphasizing the critical role of faculty development and training in SBE, this study aims to offer a framework for researchers to enhance simulation methodology training for faculty, proven successful in higher education settings. Encouragement is given for further research into SBE faculty training.
